# The Phosphor Necrosis

**Published:** 1893-06

**Authors:** 


Article VII.

### THE PHOSPHOR NECROSIS.

The phosphor necrosis, says Prof. Naumann, in *Zahnarztliches Wochenblatt*, is a disease connected with the production of phosphorous matches. The access of the phosphorous vapors to the periosteum seems to be afforded by carious teeth, and workmen with bad teeth have contracted the disease three times as often as those having sound teeth.

Usually the disease begins with toothache; an alveolar abscess is then developed, resulting in periostitis, with strong exudation of pus. The gums become swollen and abscesses are formed, whose rancid pus contains pieces of bone. The teeth fall out, the bone is deprived of the soft parts, and often shows a raw surface. General indisposition, loss of appetite, severe pains and fever bring about such debility in the patients that they soon succumb to it, if death does not occur even before through septic infection, waxy degeneration, inflammation of the kidneys, pneumonia, or the transportation of the process to the base of the skull. The favorable course is accompanied by the expulsion of the necrosed bone, whose dissolution occurs only after many years.

The prognosis is more favorable when the necrosis attacks the superior maxilla, because the abscesses forming there can find an easier outflow. The formation of the abscesses beyond the margo infraorbitalis is a sign that the process has reached the base of the skull.

The first prophylactic measure (turpentine, use of white phosphorus) proving of no effect, the chief care is then as to cleanliness; forbidding to eat in the factory rooms; change of clothes; medical examination of employes, especially with reference to carious teeth; prohibition of home industry

The therapy must aim next, at the appearance of the first symptoms, to exclude the occupation with phosphor. For the first stage, cold applications, bleeding, and antiseptic mouth washes are advisable. Then the lancing of the abscesses may be proceeded with. In the later stages it is necessary to remove the necrotic bone piece by operation, on which point opinions differ as to early or late operations. Against late operations it is argued the general condition of the patient suffers too much if we wait till the complete dissolution of the sequestor, and a portion of the new bone formation is again destroyed. On the other hand, it is objected to early operations, that the new formation of bone is not sufficient; that only a fibrous ring is formed instead of the sequestor, which brings about functional disturbance and destruction.

In observing the pathological and anatomical processes, we find periostitis with exudation. The latter is first serous, then pus-like, and lifts the periosteum from the bone, and thus leads to formation of abscesses, and at the same time also to osteophyte. The resulting alimentary disturbance has for its consequence the death of the bone and the formation of the sequester.

The author concludes with a description of a case of a patient, thirty-two years old, who had undergone an operation of the inferior maxilla for phosphorous necrosis four years before, and who, on taking to her former occupation, has again contracted the disease, this time in the superior maxilla. The operation took place April 27th; both halves of the face were much swollen; the gums were covered with a rancid pus; pus exuded on pressue from the space between the gum and the loose teeth. The alveolar process of the right upper maxilla was extracted with a bone forceps. Though the general condition was good at the beginning of the operation, the exudation of pus toward the end was still very strong. On June 6th a fluctuating tumor appeared on the infraerbital ridge of the right eye, which, being lanced two days later, exuded plenty of rancid pus. June 6th, collapse and death.

The head was preserved in alcohol, and an examination showed no new formation of bone in place of the removed portion from the inferior maxilla. The supposition that the abscess on the margo infraorbitalis points to the formation of basilar meningitis has been substantiated. This basilar meningitis must have been the cause of death.

*Items of Interest.*

